# The Role of Direct Oral Anticoagulants in Treatment of Cancer-Associated Thrombosis

**DOI:** 10.3390/cancers10080271

**Published:** 2018-08-15

**Authors:** Hanny Al-Samkari, Jean M. Connors

**Affiliations:** 1 Division of Hematology, Massachusetts General Hospital, Boston, MA 02114, USA; hal-samkari@mgh.harvard.edu; 2 Division of Hematology, Brigham and Women’s Hospital, Boston, MA 02215, USA

**Keywords:** direct oral anticoagulant, cancer-associated thrombosis, VTE, venous thromboembolism, malignancy, low molecular weight heparin, dalteparin, edoxaban, rivaroxaban

## Abstract

Venous thromboembolism (VTE) complicates the clinical course of approximately 5–10% of all cancer patients. Anticoagulation of the cancer patient often presents unique challenges as these patients have both a higher risk of recurrent VTE and a higher risk of bleeding than patients without cancer. Although low molecular weight heparins (LMWH) are the standard of care for the management of cancer-associated VTE, their use requires once or twice daily subcutaneous injections, which can be a significant burden for many cancer patients who often require a long duration of anticoagulation. The direct oral anticoagulants (DOACs) are attractive options for patients with malignancy. DOACs offer immediate onset of action and short half-lives, properties similar to LMWH, but the oral route of administration is a significant advantage. Given the higher risks of recurrent VTE and bleeding, there has been concern about the efficacy and safety of DOACs in this patient population. Data are now emerging for the use of DOACs in the cancer patient population from dedicated clinical trials. While recently published data suggest that DOACs hold promise for the treatment of cancer associated VTE, additional studies are needed to establish DOACs as the standard-of-care treatment. Many such studies are currently underway. The available data for the use of DOACs in the treatment of cancer-associated VTE will be reviewed, focusing on efficacy, safety, and other considerations relevant to the cancer patient.

## 1. Introduction

Malignancy is a known risk factor for venous and arterial thrombosis. Venous thromboembolism (VTE) occurs in approximately 5–10% of cancer patients, a 4 to 7-fold increased risk over patients without cancer [1]. It is the second leading cause of death in cancer patients [2] and occurrence of VTE increases the likelihood of death from cancer by approximately 2 to 4-fold [3,4,5]. Beyond the increased VTE risk from the malignancy itself, the treatments for cancer—cytotoxic chemotherapy, certain targeted therapies, hormonal therapy and radiation therapy—further increase risk [2]. While standard-of-care management of cancer-associated VTE for over a decade has been therapeutic anticoagulation with low molecular weight heparin (LMWH) [6], this field is rapidly evolving, with recent evidence suggesting non-inferiority of oral direct factor Xa inhibitors to prevent cancer-associated VTE recurrence [7,8]. Determination of optimal anticoagulation management in cancer patients is often difficult. While direct oral anticoagulants (DOACs) are an attractive option given their oral bioavailability, a critical analysis suggests that they may not be optimal in several cancer patient populations. The risk of bleeding may be elevated in certain tumor types, cancer-directed therapies may interact with DOAC metabolism, and advanced age and frailty in this population may increase risk of complications. In this review, we explore the challenges of anticoagulation in the cancer population, the options for treating these patients, and offer evidence-based recommendations regarding the use of DOACs in the cancer patient. 

## 2. Thromboembolic and Bleeding Risk in the Cancer Population

As the cancer patient population is markedly heterogeneous, so too is VTE risk in this population. This is a major consideration when making clinical decisions concerning the length, intensity, and type of anticoagulation in these patients. Tumor origin is important, with pancreatic ductal adenocarcinoma and gastric adenocarcinoma [9] imparting the highest risk followed by lung, gynecologic, hematologic, testicular, and bladder cancers [9,10,11]. Cancer stage impacts risk dramatically, as patients with metastatic disease have an approximately 20-fold increased risk of first VTE compared with the non-cancer population [1]. As is true for VTE in the non-cancer patients, VTE risk in the cancer population also increases with age, body mass index (BMI), and following surgery [9].

Anticoagulation of the cancer patient is complicated by both recurrent thrombosis and bleeding at higher rates than those without cancer. The rate of recurrent VTE in the cancer patient is 3 to 4-fold that of patients without cancer [12,13], occurring in approximately 20% of patients. Similarly, the rate of major bleeding in the anticoagulated cancer patient is approximately 2 to 3-fold that of the anticoagulated patient without cancer [13,14], with one large cohort reporting a 12-month major bleeding rate of 12.4% (versus 4.9% in patients without cancer) [13]. Both recurrence and bleeding rate appear to be related to cancer severity independent of under-or over-anticoagulation [13]. This finding is of significant consequence when evaluating the trials examining treatment for cancer-associated VTE, as the cancer patient population is dramatically heterogeneous and enrichment of a given trial population with high or low severity patients may have a considerable impact on the trial results.

## 3. Vitamin K Antagonists and Low Molecular Weight Heparins for Cancer-Associated VTE 

The current standard of care for management of cancer-associated VTE as recommended by numerous guidelines and professional societies is LMWH [15,16,17,18]. The body of evidence on which this recommendation is based is formed primarily by five major randomized, controlled, open-label, multicenter trials that each compared a LMWH agent to vitamin K antagonists (VKAs) in the initial management of cancer-associated VTE:

- The CANTHANOX trial compared enoxaparin 1.5 mg/kg once daily to warfarin over a 3-month treatment period in 146 patients with cancer-associated thrombosis [19]. The trial was ended early due to poor accrual. The trial used a composite major outcome of recurrent VTE or major bleeding event. There were fewer major outcome events those receiving LMWH, but this was not statistically significant (10.5% versus 21.1%, *p* = 0.09). The rate of major bleeding was 16.0% in the warfarin arm and 7.0% in the enoxaparin arm (*p =* 0.09).
- The LITE trial compared tinzaparin (175 anti-Xa units/kg once daily) with usual care of heparin transitioned to a VKA in 200 patients with cancer-associated thrombosis (PE or proximal DVT) [20]. Following the 3-month treatment period, anticoagulation was discontinued unless oral anticoagulation was indicated (as judged by the patient’s primary physician). At 3 months, 6% of patients treated with tinzaparin had recurrent VTE compared with 10% treated a VKA. At 12 months, the tinzaparin group had a significantly lower rate of recurrent VTE than the VKA group (7% versus 16%, *p* = 0.044), although not all patients remained on anticoagulation after 3 months. The rate of major bleeding was 7% in both groups.
- The ONCENOX trial randomized 102 cancer patients to receive enoxaparin 1 mg/kg once daily, enoxaparin 1.5 mg/kg once daily, or warfarin for 6 months after a 5-day enoxaparin 1 mg/kg twice daily lead-in [21]. The trial was closed early due to slow accrual. There were no significant differences in rates of recurrent VTE (6.5% of patients treated with enoxaparin and 10.0% in the VKA group) or bleeding (9.0% of patients treated with enoxaparin and 2.9% in the VKA group). *p* values were not reported for these outcomes, though the authors stated that no trends or significance could be observed due to small numbers of events.
- The CLOT trial, considered to be the most definitive because of the number of patients enrolled and duration of treatment, compared 6 months of dalteparin (200 IU/kg once daily for 1 month followed by 150 IU/kg once daily for 5 months) with 6 months of VKA therapy (following a 5–7 day dalteparin bridge) in 672 cancer patients [6]. At 6 months, the dalteparin group had a significantly lower rate of recurrent VTE than the VKA group (17% versus 9%, *p* = 0.002). There were no differences in the rates of major bleeding between the two groups (6% in the dalteparin group and 4% in the VKA group, *p* = 0.27). While the trial did not find a mortality difference in the two groups, a post-hoc analysis did find a benefit for dalteparin in patients with localized cancer (at 12 months from randomization, 20% mortality in the dalteparin group vs. 36% in the VKA group, *p* = 0.03) [22].
- The CATCH trial, published 12 years after the CLOT trial, compared 6 months of tinzaparin (175 anti-Xa units/kg once daily) with warfarin in 900 cancer patients with a life expectancy of greater than 6 months [23]. The rates of VTE recurrence (7.2% in the tinzaparin group and 10.5% in the warfarin group, *p* = 0.07) and major bleeding (2.7% in the tinzaparin group and 2.4% in the warfarin group, *p* = 0.77) were not significantly different.

While it is possible that some of the smaller trials were not adequately powered to detect the difference in recurrent VTE risk between the treatment arms, the disparate findings of CLOT and CATCH, the two largest trials, suggest that this explanation may not be adequate. Review of baseline patient characteristics from these trials (Table 1) reveals many differences. Patients in the CLOT trial had higher rates of mortality, metastatic solid tumors, and receipt of cancer-directed therapy than in CATCH (Table 1). It had been well-established that VKA therapy was challenging in the more advanced cancer patient receiving anti-cancer therapy, with lower times in therapeutic range (TTR) than the non-cancer population [24]. Treatment with chemotherapeutics that can interact with warfarin, inconsistent dietary intake of vitamin K, and nausea and vomiting presenting a barrier to swallowing pills all contribute to the increased challenge of VKA management [25]. It is possible that the disparate outcomes of CLOT and CATCH represent a failure of VKA management, known to be more challenging in more advanced cancer patients on active cancer therapy, rather than inferiority of the anticoagulant effect of VKAs. Indeed, in CLOT, most warfarin failures were in the first month of therapy during establishment of a stable dose and 37.7% of recurrent thrombotic events occurred when the INR was <2.0. The differences in severity of cancer stage and associated complications also led to a lower event rate in the VKA arm in CATCH, resulting in an underpowered study. Improved VKA management or the use of VKAs in a population more like the CATCH trial could potentially overcome these issues, as demonstrated by a published retrospective study demonstrating the equivalence of warfarin and LMWH for prevention of VTE recurrence in cancer patients cared for in a dedicated anticoagulation clinic providing support for oncologic clinicians [26]. The TTR was 59.5% for patients treated with warfarin, and bleeding rates were similar between warfarin and LMWH-treated patients. For certain populations and with close VKA monitoring, warfarin could be equivalent to LMWH in treating cancer-associated VTE, and may be the only option for those who cannot afford LMWH or DOAC therapy. Data from registries and health claims databases suggest that at least 50% of cancer patients in clinical practice have been treated with VKAs despite published guidelines [27,28]. In one analysis, VKAs appeared to do as well as LMWH at preventing recurrent VTE, although rivaroxaban appeared to be better than both [28]. In another analysis, patients switching to a VKA at 6 months had the same rate of recurrence as those remaining on LMWH [29]. More widespread use and acceptance of the use of DOACs in the cancer patient population will likely decrease the number of patients treated with VKAs.

Another consideration is the type of statistical analysis used. While the CLOT trial found a 52% relative risk reduction and 9% absolute risk reduction in the rate of recurrent VTE for dalteparin versus VKA therapy according to the Kaplan-Meier method, this analysis does not consider the competing risk of death, which is clearly sizeable in these trials [30]. A re-analysis of CLOT using the competing risk analysis of Fine and Gray found that the risk of recurrent VTE in both treatment groups was overestimated [31]. When considering the competing risk of death, LMWH still imparted a significantly lower risk of recurrent VTE but with a lower absolute risk reduction (6%).

Even as these trials established LWMH as the standard of care in cancer-associated thrombosis, numerous issues remained. Guidelines favor continuing anticoagulation indefinitely as long as active cancer remains, yet this is supported by little data and optimal duration of therapy in cancer patients remains unclear [15,16,17,18]. As an injectable agent that can result in pain, anxiety, unsightly bruising, and painful subcutaneous hematoma formation, indefinite use of LMWH presents a clear burden to patients. This burden may be judged to be excessive by patients or providers, especially in the terminally ill. An analysis of 2941 patients from a large insurer database supports these concerns, finding median treatment durations for LMWH, warfarin, and rivaroxaban for cancer-associated VTE to be 3.3, 7.9, and 7.9 months, respectively [27]. Another large database analysis of 964 cancer patients found that rates of recurrent VTE, major bleeding, and non-major bleeding were similar in patients receiving indefinite LMWH to those completing 6 months of LMWH who were then transitioned to warfarin by providers [29]. The accumulated evidence suggests poor adherence to guidelines for use of an injectable anticoagulant by patients and providers, and supports the notion that indefinite LMWH may be unnecessary. There is a need for other satisfactory options for these patients. The DOACs, if sufficiently safe and effective, would alleviate many of the issues that hinder treatment with LMWH (route of administration) and warfarin (achieving and maintaining a therapeutic level).

## 4. Direct Oral Anticoagulants for Treatment of Cancer-Associated VTE 

The direct thrombin inhibitor dabigatran and the direct factor Xa inhibitors rivaroxaban, apixaban, and edoxaban have already replaced warfarin as the preferred agents for treatment of venous thromboembolism in patients without cancer based on the results of multiple large, randomized controlled trials [32,33,34,35,36] demonstrating non-inferiority for preventing recurrent VTE. Meta-analyses of these trials have confirmed non-inferiority of the efficacy of DOACs, with lower rates of intracranial bleeding, fatal bleeding, and clinically-relevant non-major bleeding than warfarin [37]. 

Each of the pivotal trials of a DOAC versus warfarin for VTE included a small subset of cancer patients. One meta-analysis of 6 pivotal phase III trials included a subgroup analysis of those identified as cancer patients (1581 cancer patients out of a total of 27,023 enrolled patients) [37]. Those treated with DOACs had a lower VTE recurrence rate than those treated with VKAs, with similar rates of bleeding. Another meta-analysis selecting 1132 active cancer patients from these same trials found similar rates of recurrent VTE and major bleeding for DOACs and VKAs [38]. These findings may not be generalizable to the entire cancer population, however. Several of the pivotal trials excluded active cancer patients or excluded certain groups of cancer patients. No data on the types of cancer, extent of disease, or use or type of chemotherapy are available. For example, the Hokusai-VTE trial of edoxaban vs. VKAs directly excluded cancer patients in whom long-term treatment with LMWH was anticipated [33]. The most appropriate conclusions to draw from this data are that DOACs may have similar efficacy and safety as VKAs in a highly-selected cancer patient population. Similarly, a single-center prospective cohort study of 200 highly-selected patients with cancer-associated VTE treated with rivaroxaban demonstrated rates of recurrent VTE and bleeding similar to the cancer patient subgroups receiving rivaroxaban in the EINSTEIN trials [39]. As for the two meta-analyses, the results of this study may not be generalizable to the cancer population as a whole, especially those with advanced-stage disease actively receiving chemotherapy and those with complicated comorbid conditions.

The results of two multicenter, open-label, randomized, controlled trials of direct factor Xa inhibitors with LMWH for the initial therapy of cancer-associated VTE have been published:

- The Hokusai VTE Cancer trial enrolled 1050 cancer patients with acute symptomatic or incidental PE or proximal VTE to receive LMWH for 5 days followed by edoxaban 60 mg daily or dalteparin 200 IU/kg daily for one month followed by 150 IU/kg daily [7]. Patients were treated for 6–12 months on study. For the composite primary outcome of recurrent VTE or major bleeding during the 12 months after randomization (regardless of actual duration of anticoagulation), edoxaban was non-inferior to dalteparin (HR 0.97, *p* = 0.006 for noninferiority). Rates of recurrent VTE were not significantly different in each arm (7.9% in the edoxaban arm versus 11.3% in the dalteparin arm, *p* = 0.09). Rates of major bleeding were higher in the edoxaban arm (6.9% in the edoxaban arm versus 4.0% in the dalteparin arm, *p* = 0.04) Rates of clinically relevant non-major bleeding (CRNMB) were higher in the edoxaban arm (14.6% in the edoxaban arm versus 11.1% in the dalteparin arm), but this was not statistically significant. There was no difference in overall survival.
- The SELECT-D trial enrolled 406 cancer patients with acute symptomatic or incidental PE or symptomatic proximal DVT to receive rivaroxaban (15 mg twice daily for 3 weeks, then 20 mg once daily for a total of 6 months) or dalteparin (200 IU/kg daily for 1 month followed by 150 IU/kg daily for 5 months) [8]. The primary efficacy outcome of rate of recurrent VTE was lower in the rivaroxaban arm (4% versus 11%, HR 0.43, 95% CI, 0.19–0.99), while the major safety outcomes found that major bleeding was similar (6% in the rivaroxaban arm, 4% in the dalteparin arm, HR 1.83, 95% CI, 0.68–4.96), and CRNMB was significantly higher in the rivaroxaban arm (13% vs. 4%, HR 3.76, 95% CI, 1.63 to 8.69). There was no difference in overall survival.

Baseline cancer-related characteristics, such as the fraction actively receiving cancer-directed therapies and the fraction with metastatic disease (Table 2) are similar to many of the trials comparing LMWH with VKAs (Table 1), suggesting a representative active cancer population. All patients in the SELECT-D trial and approximately 98% in the Hokusai VTE Cancer trial had active cancer at the time of enrollment, a major in contrast to the small number of patients classified as having cancer in the pivotal phase III randomized controlled VTE trials [33,35,36]. These cancer-specific trials suggest that DOACs are non-inferior to LMWH for the prevention of recurrent VTE, albeit with increased bleeding risk. Two recent systematic reviews that include these two randomized controlled trials as well as several observational cohort studies support these conclusions [40,41].

Both trials reported higher rates of gastrointestinal bleeding in patients receiving DOACs. This is consistent with several prior trials of DOACs for stroke prevention in atrial fibrillation that demonstrated an approximately 1.5-fold increased risk of GI bleeding in patients receiving a DOAC compared with warfarin [42,43,44]. While the large DOAC VTE trials did not demonstrate this increased risk [37], the higher overall bleeding risk of the cancer population [13,14] may have manifest the risk more clearly in SELECT-D and Hokusai VTE Cancer. Additionally, the GI bleeding risk may be particularly high in patients with esophageal or gastric cancer. A safety analysis performed following enrollment of the first 220 patients in the SELECT-D trial noted a nonsignificant difference in major bleeding between the rivaroxaban and dalteparin arms in 19 patients with esophageal or gastroesophageal junction cancers (more detailed information regarding this difference was not published) [8]. As a result, the Data Safety Monitoring Committee recommended modification of the study protocol to exclude patients with these types of cancers.

Similarly, in a subgroup analysis of the Hokusai VTE Cancer trial, patients with GI malignancies had an increased risk of major bleeding. Randomization was stratified according to whether certain risk factors for bleeding were present, one of which was GI cancer that had been diagnosed within 6 months prior to randomization. Analyses of these patients in the safety population with GI malignancy at randomization, both by modified intention to treat (mITT) and on-treatment, revealed major bleeding in 18 of 136 treated with edoxaban and 3 of 125 treated with dalteparin, a statistically significant difference (mITT, *p* = 0.0169; on-treatment, *p* = 0.0224). Of these GI bleeds, the majority were reported to be upper GI bleeds, consistent with the safety signal recognized in the SELECT-D trial. Given the available evidence, DOAC use in patients with gastrointestinal malignancies, particularly upper GI tract malignancies, may have an unacceptably high bleeding risk; LMWH may be more appropriate for these patients. 

Other trials of DOACs for the treatment of cancer-associated VTE are under way. The CARAVAGGIO study (NCT03045406), a phase IIIb randomized, controlled, open-label trial with an estimated enrollment of 1168 participants, is an international trial comparing apixaban with dalteparin for a 6-month treatment period. The CANVAS study (NCT02744092), a pragmatic clinical effectiveness randomized open-label trial in the US with an estimated enrollment of 940 participants, compares DOAC therapy (rivaroxaban, apixaban, edoxaban, or dabigatran, by investigator’s choice) with LMWH with or without a transition to warfarin. A phase III of the safety of apixaban versus dalteparin in cancer-associated VTE (NCT02585713) has completed enrollment of 315 patients but has not yet been analyzed. These trials and others will be crucial in confirming and further defining the role of DOACs in the treatment of cancer associated VTE.

## 5. Personalization of Therapy and Future Directions 

Many questions about treatment of cancer-associated VTE remain unanswered. In addition to deciding which anticoagulant to use for acute VTE treatment, the duration of therapy required in cancer patients is a major unanswered question, with the current general consensus and guidelines suggesting continuing anticoagulation if cancer is still present after 3–6 months or in patients actively receiving treatment [17,45]. The intensity of anticoagulation needed after the acute treatment period is also unclear. With the AMPLIFY-EXT [46] and EINSTEIN CHOICE [47] trials demonstrating benefit of reduced-dose anticoagulation with apixaban or rivaroxaban beyond 6 months in the general VTE population, with only modest additional bleeding risk, many providers will look to extrapolate this data to DOAC-treated cancer patients. As many patients with cancer-associated VTE have incurable malignancy and with it a strong non-transient pro-coagulant state, it is critical that future trials address the efficacy and safety of reduced-dose extended-duration DOAC treatment in the cancer population.

While DOAC treatment may not be optimal for all cancer patients, the addition of these agents to our armamentarium for the treatment of cancer-associated VTE provides a much-needed option. Modern treatment must eschew the one-size-fits-all approach, which for over a decade has been LMWH for treatment of all cancer-associated VTE. Personalization of care for each patient is now warranted. Assessment of all of the relevant factors—concomitant systemic therapies, aversion to injectable medications, type of malignancy, and others—allows for identification of optimal cancer patient populations for each of the three primary classes of anticoagulants (Table 3). These factors can guide therapy at this time until more data are available that identify the benefits and risks of using DOACs for VTE treatment in the many different subsets of patients with cancer.

## 6. Conclusions

Anticoagulation of the cancer patient with VTE presents unique challenges, including increased risk of bleeding and VTE recurrence compared to the non-cancer patient. For over a decade, LMWH has been the established standard of care for these patients, but additional options are emerging. New data suggest that DOACs are as effective as LMWH at preventing recurrent VTE but questions about their use in subsets of cancer patients still need to be addressed, as the bleeding risk may be higher for certain groups. More than ever before, optimal treatment of cancer-associated VTE demands a personalized approach, considering the risks and benefits of each type of anticoagulant along with patient- and malignancy-specific risks and the goals of care. Although our understanding of the role of DOACs in the treatment of cancer-associated thrombosis is advancing, additional studies are needed. Many are in progress and will help to further define the optimal VTE treatment approach in the diverse cancer patient population.

## Figures and Tables

**Table 1:** Summary of randomized trials comparing VKAs with LMWH [6,19,20,21,23].

Study (Time Period)	CANTHANOX (3 Months)		LITE (12 Months)		ONCENOX (7 Months)		CLOT (6 Months)		CATCH (6 Months)	
Treatment arm	VKA	LMWH	VKA	LMWH	VKA	LMWH	VKA	LMWH	VKA	LMWH
Recurrent VTE (%)	4.0	2.8	16.0	7.0	6.5	10.0	17	9	10.5	7.2
Major bleeding (%)	16.0	7.0	7.0	7.0	2.9	9.0	4	6	2.4	2.7
Mortality (%)	22.7	11.3	47.0	47.0	32.4	32.8	39	41	32.2	34.7
Cancer therapy ^a^ (%)	69.3	76.0	NR	NR	32.3 ^b^ 32.3 ^c^	56.7 ^b^ 35.8 ^c^	76.6	78.7	55.0	50.8
Metastatic disease (%)	52.0	53.5	36.0	47.0	52.9	61.2	68.6	65.9	54.3	55.0
VKA TTR (%)	41		NR		NR		46		47	

^a^ Receiving cancer treatment either at randomization or prior to randomization. ^b^ Percent receiving chemotherapy (trial reports separate percentages for chemotherapy and radiation therapy). ^c^ Percent receiving radiation therapy. NR, not reported.

**Table 2:** Summary of randomized trials comparing DOACs with LMWH [7,8].

Study ^a^	Hokusai VTE Cancer		SELECT-D	
Treatment arm	Edoxaban	Dalteparin	Rivaroxaban	Dalteparin
Recurrent VTE (%)	6.5	8.8	4	11
Major bleeding (%)	5.6	3.2	6	4
CRNMB (%)	12.3	8.2	13	4
Mortality (%)	26.8	24.2	25	30
Cancer therapy (%)	71.6	73.1	69	70
Metastatic disease (%)	52.5	53.4	58	58

^a^ 6-month study outcomes reported for both trials.

**Table 3:** Recommendations for use of each class of anticoagulant for treatment of cancer-associated VTE.

DOAC	Optimal	Patient without GI malignancy [7,8] Low risk for major bleeding ^a^ Ease of treatment for patient is a priority [27] No strong drug-drug interactions
	Avoid	Active GI malignancy (especially esophageal, gastroesophageal junction, or gastric cancer) [7,8] History of GI bleeding [7,8] Extremes of weight (<50 kg or >150 kg) ^b^ Renal insufficiency/fluctuating renal status
LMWH	Optimal	Frequent emetogenic chemotherapy, nausea and vomiting, difficulty with oral intake Concerns for GI absorption (feeding tubes, gastric or bowel resections) [48] Drug-drug interactions with DOAC or VKA Motivated patient willing to use for extended durations [27] Known increased bleeding risk ^a^ Recurrent cancer-associated VTE while on anticoagulants ^c^ [49,50,51]
	Avoid	Strong aversion to injectable therapy [27] Renal insufficiency/fluctuating renal status Extremes of weight (<50 kg or >150 kg) ^b^
VKA	Optimal	Any situation in which close anticoagulant monitoring is necessary ^d^ or concern for absorption and metabolism Advanced chronic kidney disease Extremes of weight (<50 kg or >150 kg) ^b^
	Avoid	Lack of access to dedicated anticoagulation monitoring service with experience caring for cancer patients [26]

^a^ If DOAC reversal agent not readily available, LMWH may be preferred for patients with increased risk of bleeding at baseline; ^b^ Prescribing information for factor Xa inhibitors and LMWH recommend against use in extremes of weight, although a recent study suggests that DOACs may be appropriate for obese patients [52]; ^c^ Using twice daily dosing of enoxaparin, given at 120–125% of standard twice-daily dosing. No data for DOACs in this setting are available, and how to increase the DOAC dose with limited pill strengths is not known; ^d^ Such as need for anticoagulation in the setting of multiple prior bleeding events. Please note: This is not an exhaustive list. Anticoagulant choices may be appropriate in some patients not meeting “optimal” criteria.
